# Interaction of the GntR-family transcription factor Sll1961 with thioredoxin in the cyanobacterium *Synechocystis* sp. PCC 6803

**DOI:** 10.1038/s41598-018-25077-5

**Published:** 2018-04-27

**Authors:** Junichi Kujirai, Sato Nanba, Taro Kadowaki, Yoshiki Oka, Yoshitaka Nishiyama, Yuuki Hayashi, Munehito Arai, Yukako Hihara

**Affiliations:** 1 Graduate School of Science and Engineering, Saitama University, 255 Shimo-Okubo, Sakura-ku Saitama, 338-8570 Japan; 2 Department of Life Sciences, Graduate School of Arts and Sciences, The University of Tokyo, 3-8-1 Komaba, Meguro-ku Tokyo, 153-8902 Japan

## Abstract

Changes in the redox state of the photosynthetic electron transport chain act as a signal to trigger acclimation responses to environmental cues and thioredoxin has been suggested to work as a key factor connecting the redox change with transcriptional regulation in the cyanobacterium *Synechocystis* sp. PCC 6803. We screened for redox-dependent transcription factors interacting with thioredoxin M (TrxM) and isolated the GntR-type transcription factor Sll1961 previously reported to be involved in acclimation responses of the photosynthetic machinery. Biochemical analyses using recombinant Sll1961 proteins of wild type and mutants of three cysteine residues, C124, C229 and C307, revealed that an intramolecular disulfide bond is formed between C229 and C307 under oxidizing conditions and TrxM can reduce it by attacking C307. Sll1961 exists in a dimeric form of about 80 kDa both under reducing and oxidizing conditions. C124 can form an intermolecular disulfide bond but it is not essential for dimerization. Based on these observations, tertiary structure models of the Sll1961 homodimer and the Sll1961-TrxM complex were constructed.

## Introduction

Photosynthetic organisms perceive changes in environmental cues as the redox changes of the electron transfer components located in the photosynthetic electron transport chain and start acclimation responses to balance the photosynthetic energy supply and its consumption by various metabolic reactions^1,2^. DNA microarray analysis of the cyanobacterium *Synechocystis* sp. PCC 6803 (S.6803) with or without addition of inhibitors of photosynthetic electron transport has suggested that the redox state of components located downstream of the plastoquinone pool is critical for transcriptional regulation both under low light^3^ and high light conditions^1^.

Recently, thioredoxin (Trx), a ubiquitous small redox protein that regulates the activity of various proteins through dithiol-disulfide-exchange reactions^4^, has been highlighted as a key factor connecting the redox change of the photosynthetic electron transport chain with transcriptional regulation. We reported that a small LuxR-type transcription factor PedR (Ssl0564) interacts with Trx to achieve redox-dependent transcriptional regulation in S.6803^5,6^. Elevation of photon flux density causes activation of photosynthetic electron transport and increase in the availability of reducing equivalents at the acceptor side of photosystem I. The redox change is transmitted to PedR via interaction with reduced form of Trx, leading to a transient conformational change and inactivation of PedR. As the acclimation responses take place to mitigate the over-reduction of the photosynthetic electron transport chain, PedR returns to the active form again. This mechanism seems to enable transient induction or repression of the target genes in response to sudden changes in photon flux density.

In the heterocystous cyanobacterium *Anabaena* sp. strain PCC 7120, an ArsR-type transcription factor RexT (Alr1867) was reported as a redox-active transcriptional repressor of the *trxA2* gene (*all1866*)^7^. Binding activity of RexT to the *trxA2* promoter is lost through the formation of an intramolecular disulfide bond between two cysteine residues in the presence of H_2_O_2_, whereas the binding activity can be restored by interaction with reduced TrxA2.

There have been several reports on isolation of interacting partners of Trx in S.6803 using Trx affinity chromatography^8–10^. However, transcription factors have not been isolated so far, probably due to the technical difficulty identifying regulatory proteins with low abundance. Thus we established a new screening system to detect specific interaction between a transcription factor and Trx within *Escherichia coli* co-expression strain. We first focused on ten OmpR-type response regulators that are encoded in the S.6803 genome and identified three of them, RpaA (Slr0115), RpaB (Slr0947) and ManR (Slr1837), as new candidates of TrxM interacting partner^11^. Furthermore, we screened 29 transcription factors having cysteine residues conserved among cyanobacterial orthologs and identified two more candidates. In this study, we focused on one of them, GntR-type transcription factor Sll1961. The gene-disrupted mutant of *sll1961* has been reported to have a defect in regulation of photosystem stoichiometry under high light conditions^12^ and in phycobilisome degradation during nitrogen starvation^13^, implying contribution of Sll1961-Trx interaction to acclimation responses of the photosynthetic machinery. Biochemical analyses using recombinant Sll1961 proteins of wild-type (WT) and mutants of three cysteine residues revealed that TrxM can reduce the intramolecular disulfide bond between C229 and C307 in Sll1961. Based on these experimental results, the structure model of the Sll1961-TrxM complex was constructed.

## Results

### Interaction between Sll1961 and TrxM detected in *E*. *coli* co-expression strain

**Figure 1:**
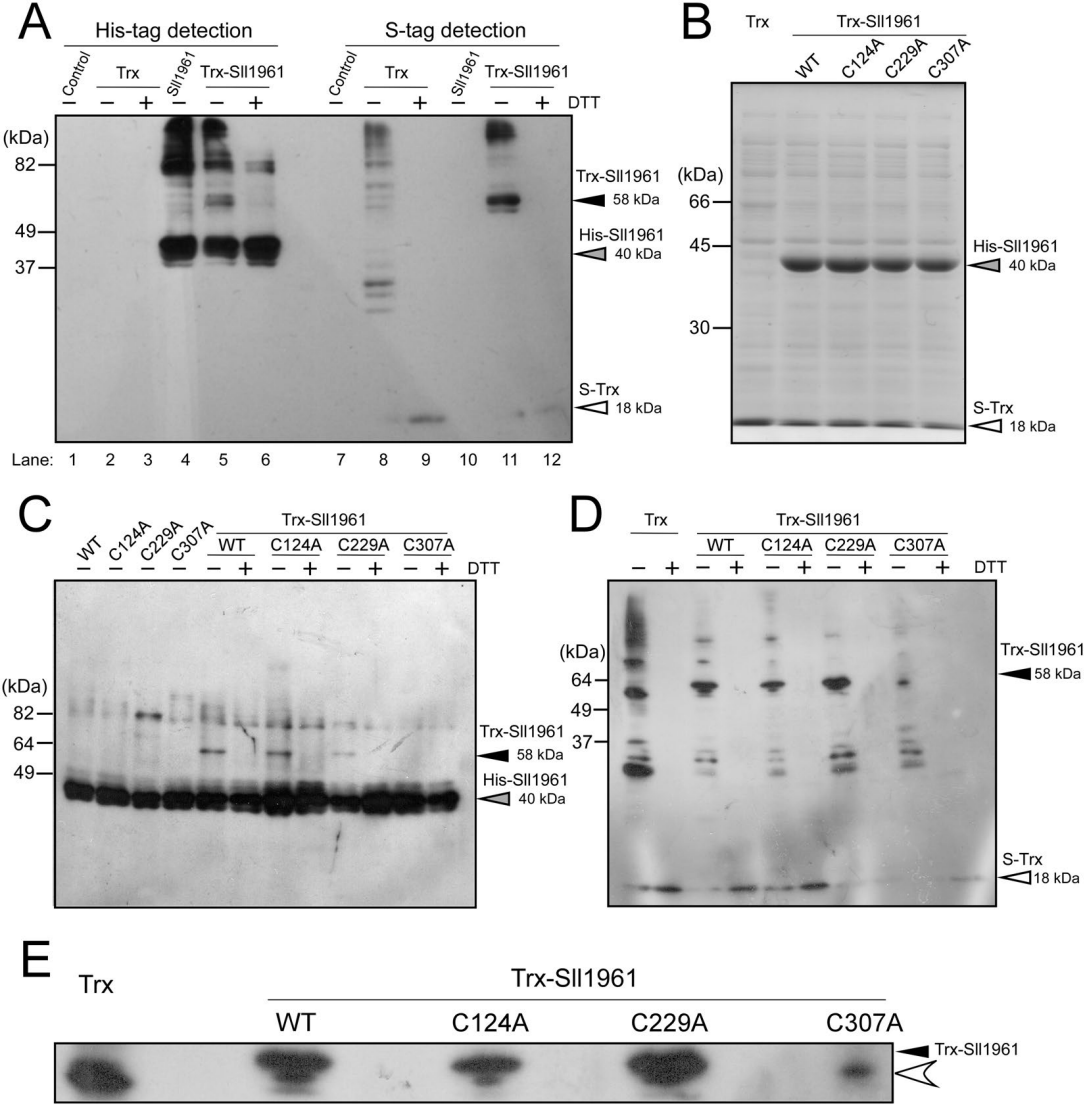
Interaction between Sll1961 and TrxM detected in *E*. *coli* co-expression strain. (**A**) Detection of the interaction between His-tagged Sll1961 and S-tagged TrxM_C35S_. After the soluble protein fraction of the control Origami2 strain (Control), the strain expressing only TrxM_C35S_ (Trx), the strain expressing only Sll1961 (Sll1961) and the strain expressing both TrxM_C35S_ and Sll1961 (Trx-Sll1961) was separated by non-reducing 12% SDS-PAGE, Sll1961 and Trx were detected by immunoblot analysis using a His-tag antibody (left) and S-protein (right), respectively. ± indicates with or without 100 mM DTT treatment. Black, gray and white arrow heads indicate the Trx-Sll1961 complex, Sll1961 monomer and Trx monomer, respectively. (**B**) Expression levels of Sll1961 and Trx in the strain expressing only TrxM_C35S_ (Trx) and strains expressing both TrxM_C35S_ and Sll1961 (WT, C124A, C229A, C307A). The soluble fraction of each strain was separated by 12% SDS-PAGE and stained with CBB. (**C**) Effect of cysteine substitutions in Sll1961 on the interaction with TrxM_C35S_. The soluble fraction of the strains expressing only Sll1961 (WT, C124A, C229A, C307A) and the strains expressing both TrxM_C35S_ and Sll1961 (WT, C124A, C229A, C307A) was separated by non-reducing 12% SDS-PAGE and Sll1961 was detected by immunoblot analysis using a His-tag antibody. (**D**) The soluble fraction of the strain expressing only TrxM_C35S_ (Trx) and the strains expressing both TrxM_C35S_ and Sll1961 (WT, C124A, C229A, C307A) was separated by non-reducing 12% SDS-PAGE and Trx was detected by immunoblot analysis using S-protein. (**E**) The enlarged view of the Trx-Sll1961 complex and the adjacent non-specific band observed in Fig. 1D.

We detected interaction between Sll1961 and TrxM using the previously established screening system^11^. In this system, a His-tagged Sll1961 (His-Sll1961) and S-tagged TrxM_C35S_ are co-expressed in *E*. *coli* Origami2 (DE3) cells carrying mutations in glutathione reductase and thioredoxin reductase genes to facilitate disulfide bonds formation in the cytoplasm. TrxM_C35S_ is a mutant TrxM protein whose active site C35 has been substituted with a serine residue. This substitution enables the formation of a stable complex between TrxM_C35S_ and the target protein, since thiol-disulfide exchange reaction is halted at the stage of the formation of the mixed-disulfide intermediate. Four different *E*. *coli* strains were used for this experiment: a control Origami2 strain; a Trx strain expressing only TrxM_C35S_; a Sll1961 strain expressing only Sll1961; and a Trx-Sll1961 strain expressing both TrxM_C35S_ and Sll1961. After induction of recombinant proteins by adding IPTG, soluble protein fractions of each *E*.*coli* strain were analyzed by non-reducing SDS-PAGE and following immunodetection. When using an anti His-tag antibody to detect His-Sll1961 protein (Fig. 1A, lanes 1–6), a 40 kDa band of the Sll1961 monomer (gray arrow) and high-molecular weight oligomers were observed in the protein extract of the Sll1961 strain (lane 4). A 58 kDa band (black arrow) was additionally detected in that of the Trx-Sll1961 strain (lane 5) but disappeared by incubation with DTT before electrophoresis (lane 6). When using S-protein to detect the S-tagged TrxM_C35S_ (lanes 7–12), many bands were detected in the Trx strain (lane 8) but they disappeared, other than a 18 kDa Trx monomer band (white arrow), following incubation with DTT (lane 9). These bands might be complexes between Trx and endogenous *E*. *coli* proteins. In the Trx-Sll1961 strain protein extract, a distinct band of 58 kDa was detected (lane 11) instead of many faint bands observed in the Trx strain, but disappeared after treatment with DTT (lane 12). The 58 kDa band, which can be detected by both anti His-tag antibody and S-protein in the absence of DTT, must be the complex of the monomeric Sll1961 and the monomeric Trx.

Sll1961 possesses three cysteine residues, C124, C229 and C307, some of which can work as interacting partners of Trx. We expected that examination of the degree of conservation of cysteine residues among Sll1961 orthologs universally present in cyanobacterial species^14^ may give us implication for the physiological significance of three cysteine residues in Sll1961. Supplementary Fig. S1 shows the phylogenetic tree of the Sll1961 orthologs in 41 representative cyanobacterial species and the plastid (cyanelle) of *Paulinella chromatophora* together with information on conservation of cysteine residues. The numerals shown in the head line are the amino acid positions in Sll1961. Marine *Synechococcus* and *Prochlorococcus* have a tendency to contain more cysteines than fresh water species. The most notable is the high conservation of C307 which exists in all marine and fresh water species except for three species in *Anabaena* and *Nostoc*. C229 was conserved in 30 species out of 42, whereas C124 was only present in S.6803. It may be possible that C124 in S.6803 is used as an alternative over C134 which is widely conserved within other species (38 species out of 42). In addition, there exist some cysteine residues well-conserved in cyanobacteria other than S.6803, such as C280 present in 31 species out of 42 and C23 highly conserved in marine species.

In order to confirm the Sll1961-Trx complex formation and to examine the contribution of each cysteine residue to the complex formation, His-Sll1961 whose cysteine residue was substituted with alanine was co-expressed with S-tagged TrxM_C35S_. Reducing SDS-PAGE revealed that the amounts of accumulated His-Sll1961 and S-Trx in the soluble protein fraction were similar among co-expression strains, Trx-Sll1961_WT_, Trx-Sll1961 _C124A_, Trx-Sll1961_C229A_ and Trx-Sll1961_C307A_ (Fig. 1B). By non-reducing SDS-PAGE followed by immunoblot analysis using an anti His-tag antibody (Fig. 1C) and S-protein (Fig. 1D,E), the 58 kDa band (black arrow) can be detected in the Trx-Sll1961_WT_, Trx- Sll1961_C124A_ and Trx-Sll1961_C229A_ strains, but not in the Trx-Sll1961_C307A_ strain. The band detected by S-protein just below the Sll1961-Trx complex was considered to be a complex of Trx and an *E*. *coli* protein (Fig. 1E, white arrowhead).

### Analysis of the Sll1961-Trx interaction using purified proteins

The experiment using *E*. *coli* co-expression strain revealed that Sll1961 interacts with TrxM and C307 of Sll1961 is essential for the interaction. To confirm this, His-Sll1961_WT_, His-Sll1961_C124A_, His-Sll1961_C229A_ and His-Sll1961_C307A_ proteins were purified from the respective single-expression strains by nickel affinity chromatography. The purified Sll1961 proteins were detected as monomer by non-reducing SDS-PAGE after treatment with 100 mM DTT (Supplementary Fig. S2). Treatment with 500 μM of thiol-specific oxidizing agent diamide resulted in detection of the faint oligomer bands, but large fractions of Sll1961 protein remained as monomer.

**Figure 2:**
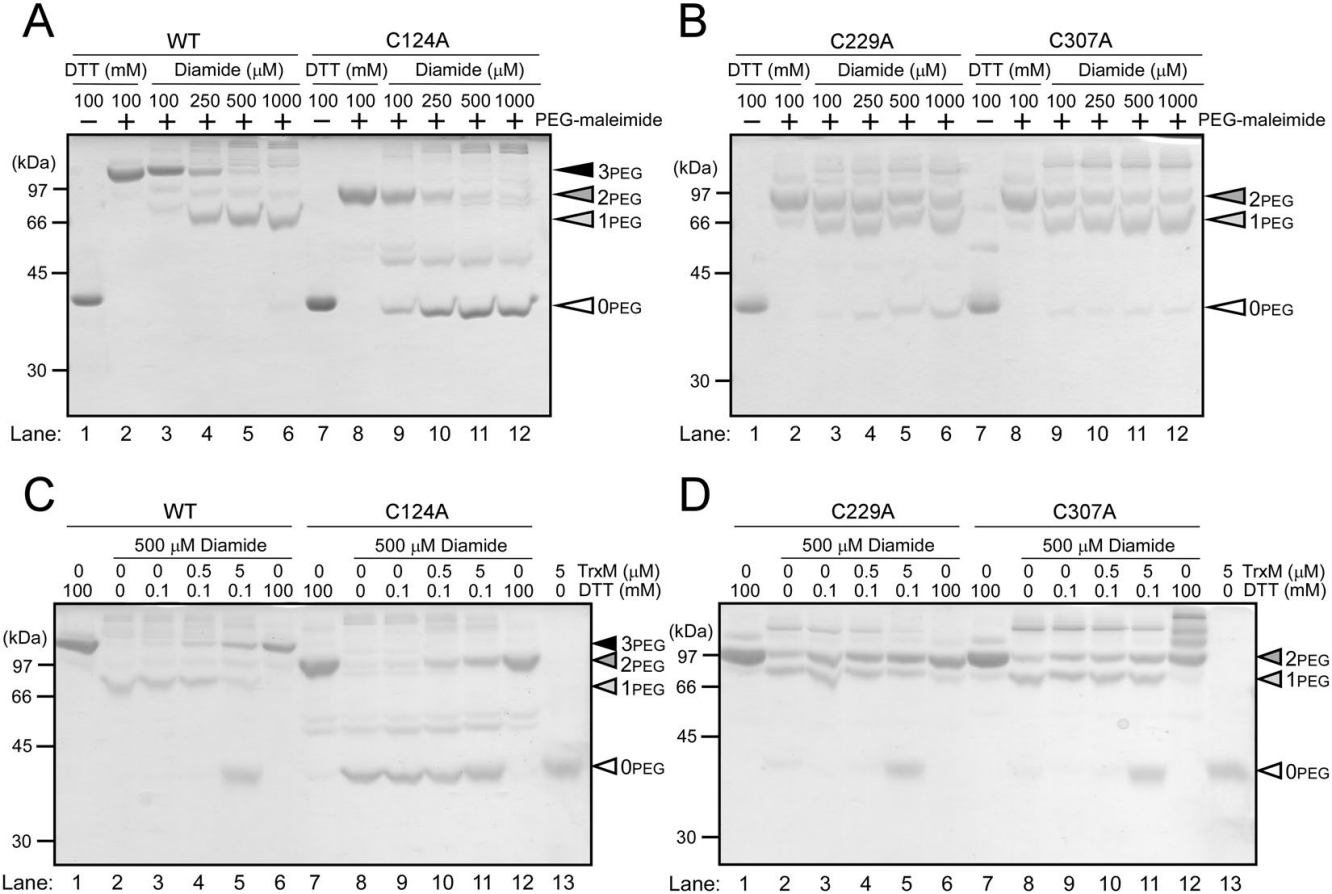
Thiol-redox state of His-Sll1961 protein detected by modification with PEG-maleimide. (**A**,**B**) WT and cysteine mutants of His-Sll1961 protein were reduced by DTT or oxidized by diamide, modified with PEG-maleimide, fractionated by non-reducing 12% SDS-PAGE and stained with CBB. (**C**,**D**) Effects of incubation with TrxM on the redox state of Sll1961. WT and cysteine mutants of His-Sll1961 protein oxidized by 500 μM diamide was incubated with DTT (0.1 or 100 mM) or TrxM (0.5 or 5 μM) in the presence of 0.1 mM DTT, modified with PEG-maleimide, fractionated by non-reducing 12% SDS-PAGE and stained with CBB. 0PEG, 1PEG, 2PEG and 3PEG indicate the number of cysteine residues modified with PEG-maleimide.

In order to examine whether three cysteine residues in Sll1961 are redox active or not, WT and cysteine mutants of His-Sll1961 protein were incubated with DTT or diamide, treated with the thiol-modifying agent PEG-maleimide (average molecular mass: 5 kDa), and then subjected to non-reducing SDS-PAGE (Fig. 2A,B). When His-Sll1961_WT_ treated with 100 mM DTT was further incubated with PEG-maleimide, a significant mobility shift was observed (Fig. 2A, lane 2) compared to the sample without incubation with PEG-maleimide (lane 1). This indicates the attachment of PEG-maleimide to all three cysteine residues (denoted as “3PEG” in Fig. 2). On the other hand, when His-Sll1961_WT_ was treated with diamide and incubated with PEG-maleimide, the 3PEG band gradually decreased and a single faster migrating band appeared in a diamide concentration-dependent manner (lanes 3–6). The faster migrating band is “1PEG”, showing that two cysteine residues were oxidized. It is of note that oxidation of all three cysteines (0PEG) or only one cysteine (2PEG) was hardly observed in His-Sll1961_WT_.

When His-Sll1961_C124A_ treated with 100 mM DTT was modified with PEG-maleimide, the attachment of PEG-maleimide to C229 and C307 resulted in band shift from 0PEG (lane 7) to 2PEG (lane 8). Oxidization with diamide resulted in decrease of the 2PEG band and increase of the 0PEG band in a diamide concentration-dependent manner (lanes 9–12). The 1PEG band was not detected, indicating oxidization of C229 and C307 proceeds simultaneously. Figure 2B shows the quite similar results on the redox change of His-Sll1961_C229A_ and His-Sll1961_C307A._ In both cases, the 2PEG band was detected after treatment with 100 mM DTT and PEG-maleimide (lanes 2 and 8). When treated with diamide, the 2PEG and 1PEG bands with similar intensity were detected irrespective of diamide concentration (lanes 3–6, 9–12), indicating coexistence of proteins in which one of two cysteine residues became oxidized or both were insensitive to oxidation. There seems no simultaneous oxidization of C124 and C307, or C124 and C229. Based on these observations, we supposed that an intramolecular disulfide bond is formed between C229 and C307 in the oxidized His-Sll1961_WT_ and this redox state was detected as 1PEG.

Next, we examined the effect of addition of TrxM to the oxidized His-Sll1961 (Fig. 2C,D). In these experiments, TrxM_WT_ but not TrxM_C35S_ was used in order to examine the native thiol-disulfide exchange reaction. Since the electrophoretic mobility of TrxM modified with PEG-maleimide (lane 13) was similar to that of Sll1961 without modification (0PEG), addition of 5 μM TrxM resulted in detection of the band at 0PEG position (lanes 5 and 11). In His-Sll1961_WT_, treatment with 500 μM diamide resulted in detection of the 1PEG band (Fig. 2C, lane 2) as observed in Fig. 2A. Although incubation with 0.1 mM DTT had no effect on this band pattern (lane 3), addition of 0.5 or 5 μM TrxM concomitantly with 0.1 mM DTT resulted in decrease in the 1PEG band and increase in the 3PEG band (lanes 4 and 5). The similar effect of TrxM was observed in the case of His-Sll1961_C124A_. Namely, incubation with 0.1 mM DTT had no effect on the 0PEG band formed by treatment with 500 μM diamide (lane 9), whereas addition of 0.5 or 5 μM TrxM concomitantly with 0.1 mM DTT resulted in decrease in the 0PEG band and increase in the 2PEG band (lanes 10 and 11). In the case of His-Sll1961_C229A_ and His-Sll1961_C307A_, the intensity of 2PEG and 1PEG bands was not affected by the addition of TrxM (Fig. 2D, lanes 3–5 and 9–11). These results clearly show that TrxM can reduce the intramolecular disulfide bond between C229 and C307 in the oxidized His-Sll1961_WT_ or His-Sll1961_C124A_, but not the oxidized cysteine in His-Sll1961_C229A_ and His-Sll1961_C307A._

### Analysis of oligomerization state of Sll1961 by size exclusion chromatography

**Figure 3:**
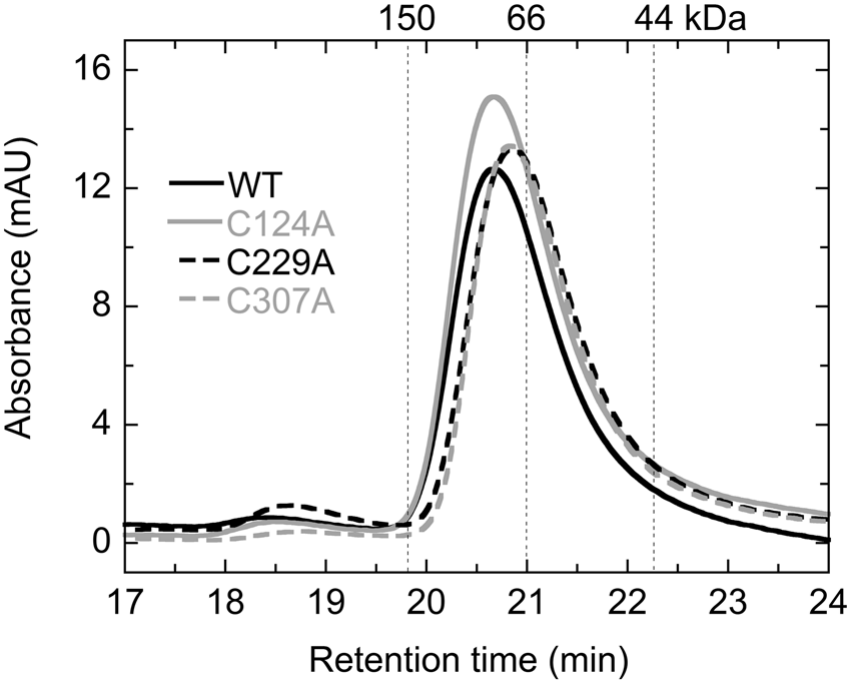
Size exclusion chromatography elution profiles of His-Sll1961 protein. WT and cysteine mutants of His-Sll1961 recombinant proteins were reduced with 100 mM DTT for 15 min at room temperature and applied to a gel filtration column. Vertical dotted lines show the retention times of standard proteins with 150, 66, and 44 kDa.

Although most GntR-family transcription factors are known to exist as a homodimer^15^, dimerization of Sll1961 has seldom been detected in our electrophoretic experiments. This may be due to the instability of the Sll1961 dimer in the presence of SDS. Thus we performed size exclusion chromatography to examine the oligomerization state of Sll1961. WT and cysteine mutants of His-Sll1961 protein were reduced with 100 mM DTT for 15 min at room temperature and filtrated before analysis. We found that all proteins eluted at the similar retention time with an estimated molecular weight of about 80 kDa (Fig. 3). The results indicate that the WT and cysteine mutants of His-Sll1961 form dimers even under reducing conditions. To determine the molecular weights more accurately, we also performed size exclusion chromatography equipped with static light scattering (Supplementary Fig. S3). The molecular weights thus obtained were 82 kDa for His-Sll1961_WT_, 80 (±1) kDa for His-Sll1961_C124A_, 78.1 (±0.1) kDa for His-Sll1961_C229A_, and 86 (±1) kDa for His-Sll1961_C307A_. These results clearly show that the WT and cysteine mutants of His-Sll1961 exist in a dimeric form even without formation of disulfide bonds.

### Analysis of disulfide bond formation in oxidized Sll1961 by mass spectrometry

**Figure 4:**
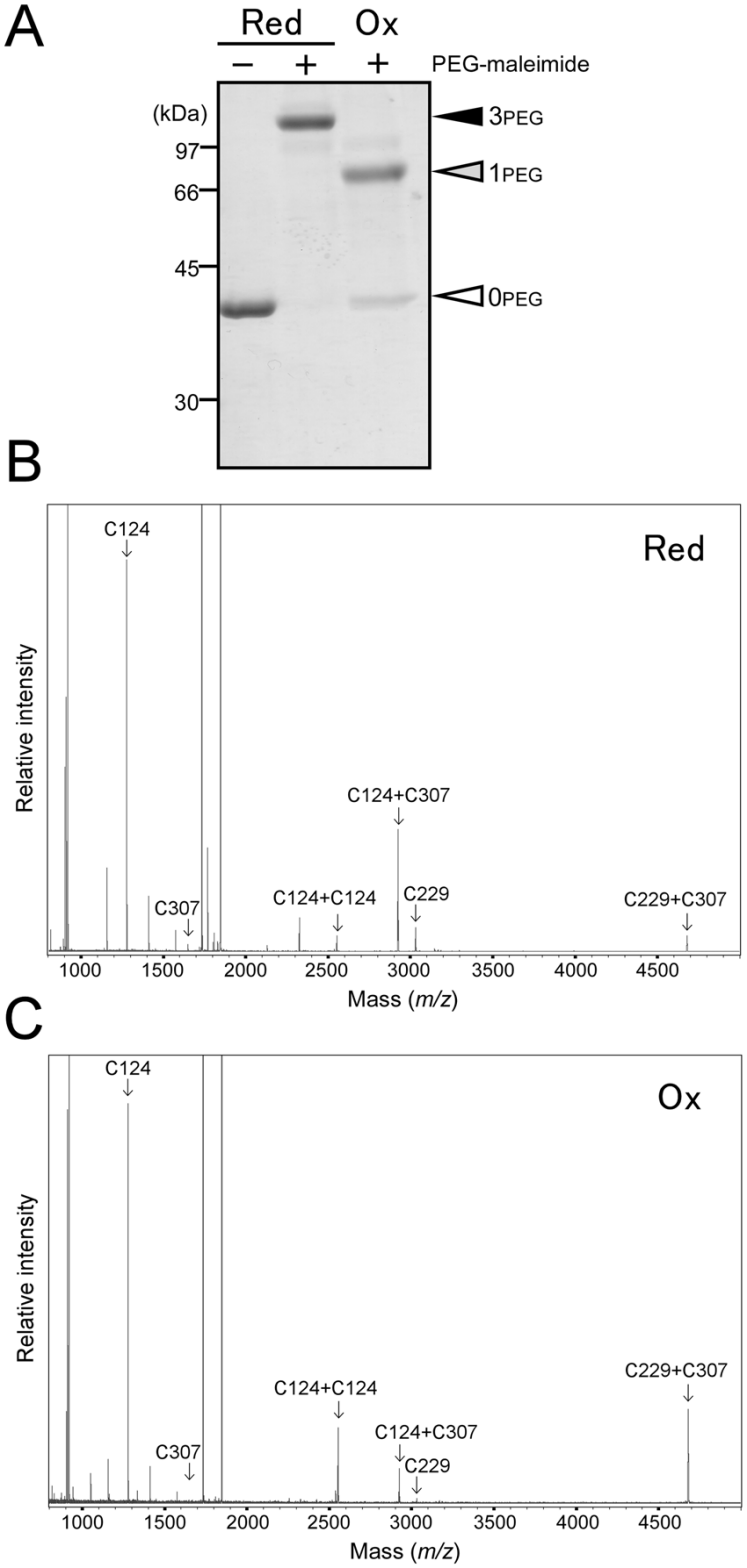
Mass spectrometry analysis of redox-treated His-Sll1961_WT_. (**A**) Thiol-redox state of reduced and oxidized samples detected by modification with PEG-maleimide. The reduced (Red) and oxidized (Ox) samples were prepared by incubation with 20 mM DTT for 15 min and with 1 mM diamide for 1 h, respectively. (**B**) MALDI-TOF mass spectra for the tryptic digests of reduced Sll1961. (**C**) MALDI-TOF mass spectra for the tryptic digests of oxidized Sll1961. Peptides containing cysteine residues were marked by arrows and labeled according to the residue number of the cysteine.

Disulfide bond formation in oxidized Sll1961 protein was further examined by mass spectrometry analysis. His-Sll1961_WT_ protein was reduced with 20 mM DTT for 15 min or oxidized with 1 mM diamide for 60 min at room temperature. Redox state of cysteine residues in these samples were examined by modification with PEG-maleimide followed by non-reducing SDS-PAGE (Fig. 4A). The reduced sample was detected as the single 3PEG band, whereas the intense 1PEG band and faint 0PEG band were observed in the case of the oxidized sample. After desalting, the redox-treated samples were digested by trypsin and matrix-associated laser desorption/ionization time-of-flight (MALDI-TOF) mass spectra were recorded (Fig. 4B,C). In the reduced sample (Fig. 4B), the tryptic fragments containing C124, C229 and C307 were detected as the peaks at *m/z* = 1277.65, 3029.79 and 1648.76, respectively. Moreover, we detected three peaks that correspond to the sum of the cysteine containing peptide fragments joined by a disulfide bond, namely, C124 + C124 (*m/z* = 2552.22), C124 + C307 (*m/z* = 2923.39) and C229 + C307 (*m/z* = 4676.83). In the oxidized sample (Fig. 4C), decrease in C124, C229 and C124 + C307 peaks and increase in C124 + C124 and C229 + C307 peaks were observed compared with the result of the reduced sample. After normalization of the data using the peak area of the tryptic fragment containing no cysteine residue (*m/z* = 1732.84), the effects of redox treatment on peak areas of cysteine-containing tryptic fragments were quantified (Table 1). These results suggested the formation of an intramolecular disulfide bond between C229 and C307 and an intermolecular disulfide bond between C124 of two monomers under oxidizing conditions. The peak that corresponds to C124 + C307 (*m/z* = 2923.39) may not be formed by a disulfide bond, since its peak area decreased upon oxidation.

**Table 1:** Effect of redox treatment on peak areas of tryptic fragments of His-Sll1961 containing cysteine residues.

Tryptic fragments containing Cys (calculated *m/z*)	Peak areas in the reduced sample (observed *m/z*)	Peak areas in the oxidized sample (observed *m/z*)^**a**^	Oxi/Red ratio
C124 (1277.46)	909 (1277.65)	827 (1277.62)	0.91
C229 (3030.62)	149 (3029.79)	9 (3030.67)	0.06
C307 (1648.91)	24 (1648.76)	ND^b^	—
C124 + C124 (2552.92)	68 (2552.22)	451 (2552.17)	6.66
C124 + C229 (4306.07)	ND	ND	—
C124 + C307 (2924.37)	730 (2923.39)	210 (2923.33)	0.29
C229 + C229 (6059.23)	ND	ND	—
C229 + C307 (4677.53)	81 (4676.83)	674 (4676.92)	8.28
C307 + C307 (3295.82)	ND	ND	—

^a^Data normalization between reduced and oxidized samples were performed using the peak area of a tryptic fragment containing no cysteine residue (*m/z* = 1732.84).

^b^ND, not detected.

### Analysis of overall structure of Sll1961 by CD and fluorescence

**Figure 5:**
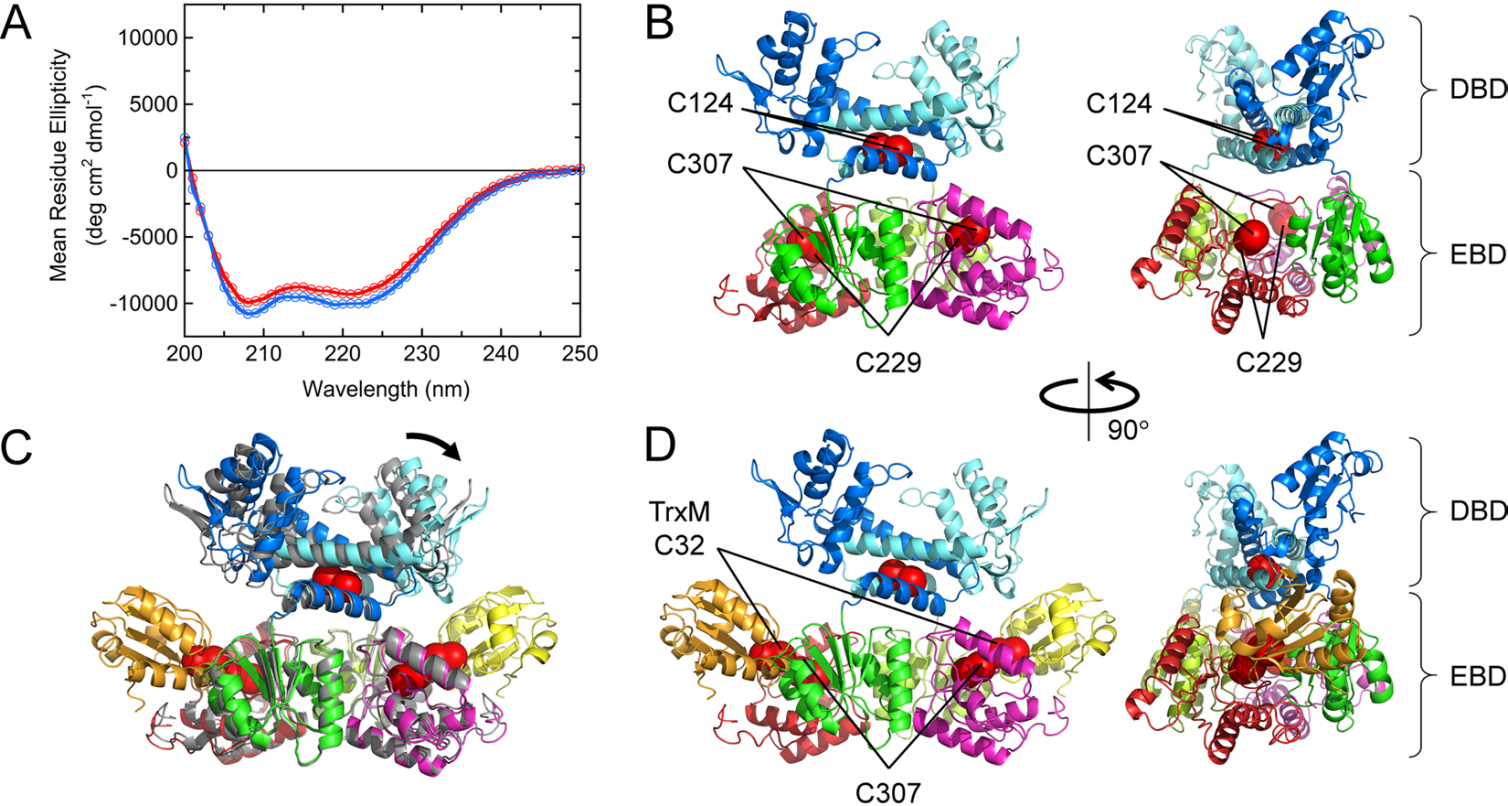
CD spectra and predicted structures of Sll1961. (**A**) Far-UV CD spectra of His-Sll1961_WT_ in the oxidized (red) and reduced forms (blue). (**B**) Predicted structure of the Sll1961 homodimer. The sulfur atoms of the cysteine residues are shown by red spheres. DBD and EBD denote the N-terminal DNA-binding domain and C-terminal effector-binding domain, respectively. (**C**) Overlaid structures of the Sll1961 homodimer (gray) and the Sll1961-TrxM complex. The black arrow denotes the direction of possible movements of the N-terminal DBD upon binding of TrxM (yellow and orange) to the C-terminal EBD of Sll1961. (**D**) Predicted structure of the Sll1961-TrxM complex, in which two TrxM molecules are bound to the Sll1961 homodimer. The figures were drawn using the PyMOL Molecular Graphics System, Schrödinger, LLC.

Far-ultra violet (UV) circular dichroism (CD) spectra of His-Sll1961_WT_ in the reduced and oxidized forms were measured to estimate their overall structures (Fig. 5A). The spectra showed two minima at 208 and 222 nm, indicating the presence of α-helical structures. In addition, intensities at 216–219 nm are similar to that at 222 nm, suggesting the presence of β-sheet structures. Indeed, the secondary structure analysis of the CD spectrum of the oxidized form using six different programs indicated that the protein contains, on average, 30 ± 5% α-helices, 20 ± 4% β-sheets, and 50 ± 2% coils (Supplementary Table S1). These results suggest that Sll1961 consists of a structural domain(s) that are classified into the α + β class, in which the secondary structure is composed of α-helices and (mostly anti-parallel) β-strands that occur separately^16^, and/or the α/β class, in which the secondary structure is composed of alternating α-helices and parallel β-strands^16^. When the protein was reduced, it contained, on average, 33 ± 4% α-helices, 17 ± 3% β-sheets, and 50 ± 2% coils, indicating that reduction of a disulfide bond in Sll1961 induces a slight increase in helical structures and a concomitant decrease in sheet structures.

Fluorescence spectrum of a single tryptophan residue (W130) of Sll1961 in the oxidized form had a peak at 344 nm (see Supplementary Fig. S4), which is close to 350 nm corresponding to the peak position of tryptophan fully exposed to solvent. This indicates that W130 is exposed to solvent. Moreover, the peak position was shifted to 347 nm by reducing the protein, indicating that W130 is more exposed to solvent in the reduced form than in the oxidized form. Since W130 is located at the N-terminal DNA-binding domain (DBD), these results suggest that reduction of Sll1961 by TrxM induces conformational changes of the N-terminal DBD, which may affect the DNA-binding ability of Sll1961.

### Structure modeling of Sll1961

The protein BLAST search^17^ of Sll1961 (residues 1–343) showed that residues 13–78 corresponds to the winged helix-turn-helix (W-HTH) DNA-binding domain of the GntR family of transcriptional regulators and that residues 3–133 corresponds to a conserved domain, having high sequence similarity with a member of the GntR family, transcriptional regulator YhcF. The GntR family of transcription factors, which was named after the gluconate operon repressor from *Bacillus subtilis*^18^, constitutes one of the most prevalent superfamilies of bacterial transcription factors. Members of the GntR family have the N-terminal DBD and the C-terminal effector-binding domain (EBD) which is also involved in dimerization^15,19^. In the DBD, the W-HTH architecture is remarkably conserved among members, although the similarity of amino acid sequence is only around 25%. Most of GntR family regulators form a homodimer and bind to either inverted or direct repeat sequences. On the other hand, the EBD shows high heterogeneity and can be classified into several subgroups, FadR, HutC, MocR, YtrA, AraR, DevA and PlmA. Structures of EBD are diverse among subgroups of GntR. For example, FadR and YtrA consist of all-α structures; HutC consists of a core of six-stranded anti-parallel β-sheet surrounded by three α-helices; AraR is composed of two subdomains, both having a core of a six-stranded parallel β-sheet flanked on each side by two α-helices; and MocR is composed of two subdomains, each having a core of a large parallel or a small anti-parallel β-sheet surrounded by α-helices^15,19,20^.

Sll1961 belongs to the PlmA subfamily, which is specific to cyanobacteria and shares very limited sequence homology with the other subfamilies^14^. Secondary structure prediction by PSIPRED^21^ showed that Sll1961 has 44% α-helices, 16% β-sheets, and 40% coils (Supplementary Fig. S5), consistent with the CD results. The prediction indicated that residues 13–78 have propensities toward three α-helices followed by two β-strands, as expected for the W-HTH motif. On the other hand, residues 97–132 have propensities to form two long α-helices, while residues 137–324 have propensities toward eight repeats of alternating β-strand and α-helix (Supplementary Fig. S4). This suggests that the C-terminal EBD of Sll1961 forms α/β structure, consistent with the CD measurement. Residues 328–343 were predicted to have random coil-like structures (Supplementary Fig. S5). These secondary structure propensities in the EBD are distinct from other GntR family members whose structures are available in the Protein Data Bank (PDB)^15,19,20^.

Because tertiary structures of Sll1961 and its orthologs are unknown^14^, we predicted the tertiary structure of the Sll1961 homodimer using bioinformatics methods (Fig. 5B; see Supplementary Text for details). Here, to construct the structure model consistent with experimental results, we took into account that an intramolecular disulfide bond is formed between C229 and C307, which is accessible to TrxM, and that C124 residues are close to each other in the homodimer. The predicted structure is composed of both the N-terminal DBD, containing the W-HTH structure, and the C-terminal EBD, which is composed of two subdomains, both having a core of ~4-stranded parallel β-sheet flanked on each side by two α-helices. This EBD structure is similar to AraR, except that the number of β-strands in the core of each subdomain is six for AraR but is four for Sll1961. The model structure suggests that Sll1961 is expected to have 50% α-helices, 14% β-sheets, and 36% coils, consistent with the secondary structure prediction and CD results. Moreover, W130 in the N-terminal DBD was 45% exposed to solvent (Supplementary Fig. S4), consistent with the fluorescence measurements. These coincidences support the validity of the model structure.

By docking TrxM to the Sll1961 homodimer, we obtained the predicted structure of the Sll1961-TrxM complex (Fig. 5D). Two TrxM molecules bind to the outer side of the homodimer and are located close to the N-terminal W-HTH domain. Comparison of the model structures of the Sll1961 homodimer and the Sll1961-TrxM complex indicates that upon TrxM binding, one of the two W-HTH domains of the Sll1961 homodimer changes its orientation to interact with TrxM (Fig. 5C). Such structural changes may affect the mutual orientation and/or the distance between the two W-HTH domains of the homodimer and, in turn, eliminate the DNA binding ability of Sll1961. These models also suggest that binding of a single TrxM molecule to the Sll1961 homodimer is sufficient to modulate the DNA binding activity of Sll1961. Solving crystal structures of Sll1961 with and without TrxM will elucidate the mechanism of Sll1961 functions.

## Discussion

Interaction between the GntR-family transcription factor Sll1961 and Trx was detected by the screening system using *E*. *coli* co-expression strain (Fig. 1A). So far, we have identified five transcription factors out of 39 as interacting partners of Trx in total. Despite existence of cysteine residue(s) in all of 39 transcription factors, only five showed interaction with Trx, suggesting that the screening system could detect only specific interaction between Trx and transcription factors. Alanine substitution experiments revealed that C307 in Sll1961 is essential for the interaction with Trx (Fig. 1C–E). It is well established that the first cysteine (C32) in the active site of Trx forms a mixed disulfide intermediate with a cysteine of the target protein and then the second cysteine (C35) attacks the mixed disulfide bond, leading to the reduction of the target protein and oxidization of Trx itself ^22^. Based on this information, we suppose that C32 of Trx interacts with C307 of Sll1961. The high degree of conservation of C307 among Sll1961 orthologs (Supplementary Fig. S1) well coincides with the biochemical data showing requirement of C307 for interaction with Trx.

Both WT and cysteine mutants of Sll1961exist in a dimeric form of about 80 kDa under reducing conditions (Fig. 3). Although oligomeric forms could be hardly observed in non-reducing SDS-PAGE (Supplementary Fig. S2), Sll1961 is likely to be a dimer in its native state and cysteine residues do not contribute to dimerization. Thiol modification (Fig. 2) and mass spectrometry (Fig. 4) analyses of the WT protein revealed that an intramolecular disulfide bond is formed between C229 and C307 under the oxidizing conditions. Although the 0PEG band, which indicates oxidation of all three cysteines, was not detected in the thiol modification experiments (Fig. 2A,C), it was observed in the oxidized sample for mass spectrometry analysis (Fig. 4A). The result of mass spectrometry analysis (Fig. 4C, Table 1) suggests that both a C229-C307 intramolecular disulfide bond and a C124-C124 intermolecular disulfide bond could be formed in the oxidized WT dimer, resulting in the detection of the 0PEG band. We think the C124-C124 intermolecular disulfide bond is non-specifically formed under oxidized conditions. It is not essential for dimer formation as suggested by size exclusion chromatography (Fig. 3) and low conservation among Sll1961 orthologs (Supplementary Fig. S1). In the case of C124A mutant, its oxidization resulted in the detection of the 0PEG, but not the 1PEG band (Fig. 2A,C). This again indicates the formation of a C229-C307 intramolecular disulfide bond. On the other hand, oxidization of C229A and C307A mutants yielded the 2PEG and 1PEG but not the 0PEG (Fig. 2B,D), which may be due to difference in sensitivity to oxidization between C124 and other two cysteines. There seem two possibilities for the origin of the 1PEG band, one is the C124-C124 intermolecular disulfide bond and other is the oxidization of one of cysteines to oxo forms such as sulfinic acid.

The incubation with TrxM resulted in the conversion from 1PEG to 3PEG in WT and from 0PEG to 2PEG in the C124A mutant (Fig. 2C) but had no effect on the 1PEG band of C229A and C307A mutants (Fig. 2D). This indicates that TrxM can reduce the C229-C307 intramolecular disulfide bond, but not other oxidized forms of cysteines. The predicted structure of the Sll1961-TrxM complex (Fig. 5D) implies that interaction with TrxM causes structural changes of the W-HTH domain of Sll1961, resulting in changes in DNA binding activity.

It has been reported that binding of effector molecules to EBD affects the conformation of DBD to regulate the DNA binding activity in the GntR-family transcription factors. For example, DNA binding of the FadR repressor regulating fatty acid metabolism in *E*. *coli* is specifically inhibited by binding of long chain fatty acyl-CoA compounds to EBD^23^. Similarly, DNA binding of AraR repressor regulating arabinose metabolism in *B*. *subtilis* is inhibited by binding of arabinose to EBD^24^.

To date, there have been two reports on the functional characterization of Sll1961 orthologs in cyanobacterial species other than S.6803. In the filamentous cyanobacterium *Anabaena* sp. PCC 7120, null mutants of *plmA* (all1076) were reported to be unable to maintain the copy number of several endogenous plasmids and exogenously introduced shuttle vector^25^. Recently, Sll1961-ortholog in *Synechococcus elongatus* PCC 7942 was isolated by yeast three-hybrid screening as an interacting factor with PII-PipX complex involved in nitrogen regulatory network^14^. Authors suggested that activity of the transcription factor can be regulated in response to 2-oxoglutarate and ATP/ADP signals through interaction with PII-PipX complex.

Although none of the reports on GntR-family regulator in heterotrophic bacteria provide clues to the function of EBD of PlmA subfamily, the report on interaction with PII-PipX in *Synechococcus elongatus* and our findings on interaction with Trx in S.6803 imply the possibility that the conformational change of EBD through protein-protein interaction resulted in changes in DNA binding activity. By the characterization of *sll1961*-disrupted mutant in S.6803, it has been suggested that Sll1961 is involved in regulation of photosystem I content under high-light conditions^12^ and phycobilisome content under nitrogen-limited conditions^13^. Under these conditions, Sll1961 may participate in transcriptional regulation in response to photosynthetic activities through interaction with Trx and to 2-oxoglutarate level through interaction with the PII-PipX complex.

## Methods

### Bacterial strains and growth conditions

*E*. *coli* XL1-blue cells were used for routine cloning and Origami2 (DE3) cells (Merck Millipore, Darmstadt, Germany) for protein expression. Bacterial cultures were grown in TB or 2xYT medium at 37 °C. When necessary, antibiotics were added at the following concentrations: ampicillin (100 μg ml^−1^), kanamycin (20 μg ml^−1^) and spectinomycin (20 μg ml^−1^).

### Construction of *E*. *coli* strains expressing His-tagged Sll1961 and/or S-tagged TrxM_C35S_

The coding region of *sll1961* was amplified by PCR using the primer set 1961-F (5′-AACATATGCTACAGTTCCAAATT-3′) and 1961-R (5′-AAGGATCCTTAAGCCGTGGCCACTTT-3′). The underlining indicates the recognition sequences of *Nde*I and *Bam*HI, respectively. PCR products were cloned into the pT7Blue T-vector (Merck Millipore), excised from the pT7Blue vector with *Nde*I and *Bam*HI and subcloned into the same restriction sites in the pET28a vector (Merck Millipore) to express proteins with an N-terminal 6xHis-tag. C124A, C229A and C307A mutations were introduced into the *sll1961* coding sequence in the pET28a vector by PCR using a KOD-plus-mutagenesis kit (TOYOBO, Osaka, Japan) with the following primer sets: C124A-F (5′-GCTTTGGAAACCATTGATTGGC-3′) and C124A-R (5′-AAGCTCTTTCACTTGGGAGA-3′), C229A-F (5′-GCCTTAGGCATTGTGAGCCTTAGC-3′) and C229A-R (5′-GGAATTCTCTGGCAGAGCTT-3′) and C307A-F (5′-GCCTCTGAACATTACATTGGCGAA-3′) and C307A-R (5′-AATAATTTCCGGCATCCGGA-3′). The underlining indicates the substitution from cysteine- to alanine-codon. Each expression construct was transformed into Origami2 (DE3) competent cells to yield the WT and cysteine mutants of the Sll1961 strains.

Cloning of the coding region of *trxM* (*slr0623*) into the pT7Blue T-vector, introduction of C35S mutation and subcloning into the pCDFDuet-1 vector (Merck Millipore) to express TrxM_C35S_ with a C-terminal S-tag were performed as described in Kadowaki *et al*.^11^. The resulting construct was transformed into Origami2 (DE3) competent cells and competent cells of the Sll1961 strains described above, to yield the Trx strain and the Trx-Sll1961 strains, respectively.

### Detection of the interaction between S-tagged TrxM_C35S_ and His-tagged Sll1961 in *E*. *coli* cells

*E*. *coli* Origami2 (DE3) strains, harboring each expression construct, were precultured in 2 ml of the TB medium containing kanamycin (for the pET28a vector) or spectinomycin (for the pCDFDuet-1 vector) at 37 °C overnight. The preculture of each strain was inoculated into 4.5 ml of the 2xYT medium to OD_600_ = 0.1 and expression of Sll1961 and TrxM_C35S_ was induced by addition of 100 μM isopropyl-D-thiogalactopyranoside (IPTG) at the mid-log phase. The cells were allowed to grow for an additional 3 h at 37 °C and harvested by centrifugation at 5,800 *g* for 2 min. Cell pellets were resuspended in the binding buffer (20 mM sodium phosphate, pH 7.4, 500 mM NaCl, 20 mM imidazole) and disrupted by sonication with Sonifier 450 (Branson, Danbury, USA) for 2 min, with two pauses of 1 min each on ice. The cell lysate was centrifuged at 16,000 *g* for 5 min and the supernatant was subjected to SDS-PAGE on non-reducing 12% gels, blotted onto polyvinylidene difluoride membrane (Immobilon-P; Millipore, Billerica, USA), and probed with rabbit polyclonal antibodies to His-tag (Bethyl, Montgomery, USA) or S-protein HRP conjugate (Novagen). The bound antibodies were detected using goat anti-rabbit IgG secondary antibodies conjugated to horse radish peroxidase (Bio-Rad, Hercules, USA) and the chemiluminescence detection reagent, EzWestLumi plus (Atto, Tokyo, Japan).

### Expression and purification of His-Sll1961 proteins from *E*. *coli* cells

His-tagged Sll1961 proteins were purified from the WT and cysteine mutants of Sll1961 strains. The preculture of each strain was seeded into 50 ml of 2xYT medium and expression of Sll1961 was induced by addition of 100 μM IPTG at the mid-log phase. The cells were allowed to grow for an additional 3 h at 37 °C and harvested by centrifugation. All protein purification procedures were performed at 4 °C. Cells were disrupted by sonication on ice in 1.5 ml of the binding buffer and the lysate was centrifuged at 16,000 *g* for 5 min. The resulting supernatant was added to a 400 μl of Ni^2+^-Sepharose resin (COSMOGEL His-Accept; Nacalai tesque, Kyoto, Japan) pre-equilibrated with the binding buffer. After rotation for overnight at 4 °C, the sample was centrifuged at 1,500 *g* for 5 min and the resin was resuspended in 1.5 ml of the binding buffer. This washing step was repeated four times with increasing concentration of imidazole (20–40 mM). Proteins were then eluted from the resin with 200 μl of the elution buffer (20 mM sodium phosphate, pH 7.4, 500 mM NaCl, 300 mM imidazole) by incubation at 4 °C for 15 min. The supernatant was collected, and the elution step was repeated once. Purified His-Sll1961 was desalted by a PD MiniTrap G-25 column (GE Healthcare, Chicago, USA) that had been equilibrated with 20 mM sodium phosphate, pH 7.4. The eluate was added with 10% (v/v) glycerol, frozen in liquid N_2_ and stored at −80 °C before use.

Protein concentration was determined using Bio-Rad Protein Assay Kit (Bio-Rad) with bovine serum albumin as the standard. The purity of the proteins was assessed by fractionating an aliquot on an SDS-PAGE gel and staining with Coomassie Brilliant Blue (CBB).

### Redox treatments of His-Sll1961 and modification of thiol groups of cysteine residues

Oxidization of His-Sll1961 (5 μM) was performed by incubation with 500 μM diamide for 60 min at room temperature. Reduction of His-Sll1961 (5 μM) was performed by incubation with 100 mM DTT or 100 μM DTT in the presence or absence of the WT TrxM protein (0.5μM or 5 μM) for 15 min at room temperature. After the redox treatments, proteins were precipitated with 10% (w/v) trichloroacetic acid, washed with ice-cold 20% acetone and the thiol groups of cysteine residues were then modified by incubation with 4 mM methoxypolyethylene glycol (PEG) maleimide (Nihon Yushi, Tokyo, Japan) at 4 °C overnight. Modified proteins were separated by non-reducing SDS-PAGE and stained with CBB.

### Mass spectrometry analysis

25 μg of His-Sll1961 protein was oxidized with 1 mM diamide for 60 min or reduced with 20 mM DTT for 15 min at room temperature. After desalting using Zeba Spin Desalting Column (Thermo Fisher, Waltham, USA) and protein quantification using Bio-Rad Protein Assay Kit, samples were subjected to digestion by Sequencing Grade Modified Trypsin (Promega, Fitchburg, USA) at a ratio of 20:1 (w/w) for 2 h at 37 °C. After desalting with ZipTip (Millipore), 1 μl of the sample was mixed with 4 μl of matrix solution of α-cyano-4-hydroxy cinnamic acid and spotted on the sample plate. MALDI-TOF mass spectra were acquired with Autoflex II (Bruker, Bremen, Germany). Calibration was performed using a Peptide Calibration Standard (Bruker) spotted on the target position next to the sample.

### Size exclusion chromatography

Size exclusion chromatography was performed using high performance liquid chromatography (HPLC) system (LP-20AP, Shimadzu, Kyoto, Japan). WT and cysteine mutants of His-Sll1961 protein were reduced with 100 mM DTT for 15 min at room temperature, concentrated to 60 μM using Amicon Ultra-0.5 mL (MWCO 10 kDa, Millipore), centrifuged at 20,600 *g* for 30 min at 4 °C, and filtrated using a 0.45 μm syringe filter (Millipore) before injection. Protein samples were injected into gel filtration columns, Superdex 75 increase 3.2/300 or Superdex 200 increase 3.2/300 (GE Healthcare), using a sample loop of 20 μl and eluted with the running buffer containing 20 mM sodium phosphate (pH 7.4), 150 mM NaCl and 3 mM DTT. The flow rate of the running buffer was 0.1 ml min^−1^. Absorbance of eluted samples was monitored at 280 nm by the UV-VIS detector (SPD-20A, Shimadzu). Alcohol dehydrogenase (150 kDa), bovine serum albumin (BSA, 66 kDa), and ovalbumin (44 kDa) were used as the standards for the Superdex 200 increase 3.2/300 column.

Determination of molecular weights was carried out on the HPLC equipped with a Viscotek TDA 305 light scattering detector (Malvern Instruments, Malvern, UK) using the Superdex 75 increase 3.2/300 column. BSA was used as a standard for calibration of the static light scattering analysis. Data analysis for estimating molecular weights was performed with the OmniSEC software (Malvern). Errors in molecular weights were estimated from duplicate or triplicate measurements.

### CD and fluorescence measurements

Far-UV CD spectra of His-Sll1961_WT_ in the reduced and oxidized forms were measured in the buffer containing 20 mM sodium phosphate (pH 7.4) and 150 mM NaCl with and without 3 mM DTT, respectively, at 0.2 mg ml^−1^ protein concentration adjusted using Amicon Ultra-0.5 mL (MWCO 10 kDa, Millipore). CD spectra were obtained on a J-805 spectropolarimeter (JASCO, Tokyo, Japan) by scanning from 250 nm to 200 nm in a quartz cuvette with a path length of 1 mm. The temperature for measurement was maintained at 25 °C by a thermostat circulating water bath. Mean residue ellipticity was calculated as described previously^26^, assuming dimer formation of the protein. Fluorescence spectra of His-Sll1961_WT_ were measured as described in Supplementary Text.

### Structure modeling

The procedure for modeling tertiary structures of Sll1961 and the Sll1961-TrxM complex is summarized in Supplementary Fig. S6 and described in detail in Supplementary Text and Supplementary Table S2. Three-dimensional structure predictions were performed using the I-TASSER server^27^. Docking predictions were performed using the ZDOCK server^28^. Foldit Standalone^29^ was used for manual refinement of predicted structures.

## Electronic supplementary material

Supplementary text, tables and figures
