# Supplementary material for: Interaction of the GntR-family transcription factor Sll1961 with thioredoxin in the cyanobacterium Synechocystis sp. PCC 6803
Source: Sci Rep. 2018 Apr 27;8:6666. doi: 10.1038/s41598-018-25077-5 (PMC5923263; doi:10.1038/s41598-018-25077-5)
Supplement: Supplementary file 1 — Supplementary text, tables and figures [file 41598_2018_25077_MOESM1_ESM.pdf]

Supplementary Information for:

Interaction of the GntR-family transcription factor Sll1961 with thioredoxin in the cyanobacterium *Synechocystis* sp. PCC 6803

Junichi Kujirai[1], Sato Nanba[1], Taro Kadowaki[1], Yoshiki Oka[2], Yoshitaka Nishiyama[1], Yuuki Hayashi[2], Munehito Arai[2], and Yukako Hihara[1]

[1]Graduate School of Science and Engineering, Saitama University, Saitama, Japan

[2]Department of Life Sciences, Graduate School of Arts and Sciences, The University of Tokyo, Tokyo, Japan

- Supplementary Text
- Supplementary Table S1 Secondary structure contents of Sll1961 estimated from the CD spectra.
- Supplementary Table S2 Top threading templates used by I-TASSER.
- Figure S1 Conservation of cysteine residues in Sll1961 orthologs.
- Figure S2 Redox treatment of WT and cysteine mutants of His-Sll1961 recombinant proteins.
- Figure S3 Elution profiles of size exclusion chromatography equipped with static light scattering.
- Figure S4 Fluorescence spectra of Sll1961.
- Figure S5 Secondary structure prediction of Sll1961.
- Figure S6 Procedure for modeling tertiary structures of Sll1961 and the Sll1961-TrxM complex.

Supplementary Text

Fluorescence measurement

Fluorescence spectra of His-Sll1961$_{WT}$ in the reduced and oxidized forms were measured in the buffer containing 20 mM sodium phosphate (pH 7.4) and 150 mM NaCl at 0.01 mg ml^{-1} protein concentration with and without 3 mM DTT, respectively. Fluorescence spectra were obtained on a Shimadzu RF-5300PC spectrofluorometer (Shimadzu, Kyoto, Japan) in a quartz cuvette with a path length of 1 cm. The temperature for measurement was maintained at 25°C by a thermostat circulating water bath. The excitation wavelength was 280 or 295 nm.

Structure modeling

The procedure for modeling tertiary structures of Sll1961 and the Sll1961-TrxM complex is summarized in Supplementary Fig. S6. Three-dimensional structure predictions were performed using the I-TASSER server[27]. Structures predicted by the RaptorX server[30] were essentially the same as those obtained by I-TASSER. Structure prediction of a whole Sll1961 molecule gave poor results especially in the EBD. To increase modeling accuracy, we split the amino acid sequence into the N-terminal W-HTH domain (residues 1-135) and the C-terminal EBD (residues 136-343), because the structure prediction methodology has been optimized for modeling single-domain proteins[27]. Structure prediction of the N-terminal domain gave good results (confidence score (C-score) = 0.61 and template modeling score (TM-score) = 0.80); typically, C-score and TM-score of larger than –1.5 and 0.5, respectively, are considered as good prediction[27] (see Supplementary Table S2 for the top templates used in the I-TASSER predictions, and the alignments and statistics for the templates). The predicted structure was similar to the crystal structure of transcriptional antiterminator from *Listeria monocytogenes* (PDB ID 4ham). Docking prediction by the ZDOCK server[28] gave a homodimer of the N-terminal domain, in which C124 was in close proximity with each other.

Structures of the C-terminal EBD predicted by I-TASSER with and without a restraint of a disulfide bond between C229 and C307 had the C-score of less than –3.5. Among the top predicted models, Model 1 had a TIM-barrel structure but had a feature inconsistent with experimental results: C307 was buried inside the protein molecule and could not be accessible to TrxM. On the other hand, Model 2 had two subdomains, both having a core of parallel β-sheet flanked on each side by two α-helices. This structure was similar to that predicted by RaptorX. However, the distance between C229 and

C307 was larger than 10 Å. To increase modeling accuracy, we again split the amino acid sequence into the N-terminal half (residues 136-222) and C-terminal half (residues 223-327) of the EBD. The C-terminal disordered region (residues 328-343) was not included here. Remarkably, structure prediction of the N-terminal half of the EBD was reliable (C-score = –0.97 and TM-score = 0.59), and the structure had a core of four-stranded parallel β-sheet flanked on each side by two α-helices. Similarly, structure prediction of the C-terminal half of the EBD with a restraint of a disulfide bond between C229 and C307 was reliable (C-score = –1.85, which is close to –1.5, and TM-score = 0.49, which is close to 0.5), and the structure had a similar structure with the N-terminal half of the EBD and maintained the C229-C307 disulfide bond. Although hydrogen bonds between the parallel β-strands were not well formed in the model structure, probably to accommodate the disulfide bond, the overall similarity with the structure of the N-terminal half of the EBD suggests that the C-terminal half of the EBD is expected to have a core of four-stranded parallel β-sheet flanked on each side by two α-helices. Then, the structures of the N- and C-terminal halves of the EBD and that of the C-terminal disordered region (taken from the predicted structure of residues 136-343) were connected using the Foldit Standalone, an interactive graphic interface for protein structure manipulation using Rosetta[29]. Here, orientations of the N- and C-terminal halves of the EBD were adjusted to make C307 exposed to solvent for TrxM binding. Docking prediction by the ZDOCK server gave a homodimer of the EBD. Finally, the homodimer of the N-terminal W-HTH domain and the homodimer of the C-terminal EBD were connected using Foldit Standalone, resulting in the three-dimensional structure model of the Sll1961 homodimer. The model structure suggests that Sll1961 is expected to have 50% α-helices, 14% β-sheets, and 36% coils, if the putative β-sheet in the C-terminal half of the EBD is included in counting the β-sheet contents.

The TrxM structure was predicted by I-TASSER (C-score = 1.10 and TM-score = 0.86). The structure of the Sll1961-TrxM complex, i.e., the hetero-tetramer composed of the Sll1961 homodimer and two TrxM subunits, was predicted by ZDOCK and manually refined using Foldit Standalone.

Supplementary Information Additional References

30. Kallberg, M. *et al.* Template-based protein structure modeling using the RaptorX web server. *Nat. Protoc.* **7**, 1511-1522 (2012).

31. Sreerama, N., & Woody, R.W. Estimation of protein secondary structure from circular dichroism spectra: comparison of CONTIN, SELCON, and CDSSTR

methods with an expanded reference. *Anal. Biochem*. **287**, 252-260 (2000).

32. Louis-Jeune, C., Andrade-Navarro, M.A., & Perez-Iratxeta, C. Prediction of protein secondary structure from circular dichroism using theoretically derived spectra. *Proteins. Structure, Function, and Bioinformatics* 80, 374-381 (2011).
33. Micsonai, A. *et al.* Accurate secondary structure prediction and fold recognition for circular dichroism spectroscopy. *Proc. Natl Acad. Sci. U S A* **112**, E3095-E3103 (2015).
34. Whitmore, L., Mavridis, L., Wallace, B.A., & Janes, R.W. DichroMatch at the protein circular dichroism data bank (DM@PCDDB): A web-based tool for identifying protein nearest neighbors using circular dichroism spectroscopy. *Protein Sci.* **27**, 10-13 (2018).
35. Lakowicz, J.R. *Principles of Fluorescence Spectroscopy*. 3rd Edition (Springer, 2006).

Supplementary Table S1 Secondary structure contents of Sll1961 in the oxidized and reduced forms estimated from the CD spectra

	Oxidized form			Reduced form		
Programs	Helix (%)	Sheet (%)	Coil (%)	Helix (%)	Sheet (%)	Coil (%)
CDSSTR [31]	32.8	18.1	49.1	36.1	16.5	47.4
CONTIN [31]	32.3	17.1	50.6	34.3	15.5	50.2
SELCON [31]	29.0	21.0	50.0	35.8	16.0	48.0
K2D3 [32]	24.8	23.5	51.7	26.8	21.6	51.6
BESTSEL [33]	22.7	24.2	53.1	29.1	19.8	51.1
PCDDB [34]	38.4	13.0	48.6	38.4	13.0	48.6
average ± SD	30 ± 5	20 ± 4	50 ± 2	33 ± 4	17 ± 3	50 ± 2

Secondary structure contents estimated using six different programs, and their average and standard deviation (SD) are shown.

Supplementary Table S2   Top threading templates used by I-TASSER

(a) TrxM

PDB	*Iden1*	*Iden2*	*Cov*	*Z*	Alignment
2yj7A	0.63	0.62	0.98	3.75	--SVIEVTDENFEQEVLKSDKPVLVDFWAPWCGPCRMIAPIIEELAKEYEGKVKVVKVNVDENPNTAAQYGIRSIPTLLLFKNGQVVDRLVGAQPKEALKERIDKHL
3f8uA	0.30	0.30	0.98	1.13	-SDVLELTDDNFESRISDTAGLMLVEFFAPWCGHAKRLAPEYEAAATRLKGIVPLAKVDCTANTNTCNKYGVSGYPTLKIFRDGEEAGAYDGPRTADGIVSHLKKQ-
4ba7A	0.60	0.57	0.95	2.75	-----SINDENFEEEVLKSDKPVLVDFWAPWCGPCRMIAPIIEELAEEYEGKVKFAKVNVDENPETAAKYGIMSIPTLLLFKNGEVVDKLVGARPKEALKERIEKHL
2i4aA	0.49	0.49	1.00	2.72	SEHTLAVSDSSFDQDVLKASGLVLVDFWAEWCGPCKMIGPALGEIGKEFAGKVTVAKVNIDDNPETPNAYQVRSIPTLMLVRDGKVIDKKVGALPKSQLKAWVESAQ
Amino acid sequence of TrxM					MSATPQVSDASFKEDVLDSELPVLVDFWAPWCGPCRMVAPVVDEISQQYEGKVKVVKLNTDENPNTASQYGIRSIPTLMIFKGGQRVDMVVGAVPKTTLASTLEKYL

PDB shows the PDB ID (4 characters) plus chain ID (1 character) of the template.

Iden1 is the percent sequence identity of the template in the threading aligned region with the query sequence.

Iden2 is the percent sequence identity of the whole template chain with the query sequence.

Cov is the coverage of the threading alignment and is equal to the number of aligned residues divided by the length of the query sequence.

Z is the normalized *Z*-score of the threading alignments. Alignment with a $Z > 1$ means a good alignment. Although the *Z*-scores are less than 1 for the predictions of residues 136-222 and residues 222-327 of Sll1961, the finally predicted structures have high C- and TM-scores showing that the predictions are reliable.

(b) Residues 1-135 of Sll1961

PDB	*Iden1*	*Iden2*	*Cov*	*Z*	Alignment
4hamA	0.22	0.25	0.96	2.79	SNAFTINTKSQLPIYEQIVQKIKEQVVKGVLQEGEKILSIREFASRIGVNPNTVSKAYQELERQEVIITVKGKGTFIANQTDK------LSSPKKLAETRTKLKETILDLVYLGVNIEEIHKLADEYSQDIIGGD
2ek5A	0.24	0.22	0.84	1.40	-----------VPLYKQIASLIEDSIVDGTLSIDQRVPSTNELAAFHRINPATARNGLTLLVEAGILYKKRGIG-FVSAQAPALIR-----ERRDAAFAATYVAPLIDESIHLGFTRARIHALLDQVAESR----
3c7jA	0.16	0.15	0.91	1.89	LSVLGNEQPPAHLARTVIEEKLRNAIIDGSLPSGTALR-QQELATLFGVSR-PVREALRQLEAQSLLRVETHKGAVVAPLITE--------DAVDAYALRILLESEALRLSIPLLDADDLAAAASYIENR-FHL-
Amino acid sequence of Sll1961					MLQFQIQNDSEIPASKQLFDQIRFAIASRQYHPGHRLPSTRQLAMMTGLHRNTISKVYQNLEDAGLVESIAGSGIYVKAPSTEEGMILDGPLFREYPEASQLIQKTIDELLGQGLNLSQVKELCLETIDWRLRST

(c) Residues 136-222 of Sll1961

PDB	*Iden1*	*Iden2*	*Cov*	*Z*	Alignment
2n3zA	0.25	0.21	0.93	0.74	GRLVVVVTS------EQLKEEVRKKFQVEVRLVTEEDAKQVIKEIQKKKVVLVGVSEKLLQKIKQEANVQVYRVTSNDLEQVVKDVK
4mgeA	0.21	0.21	0.94	0.62	MNILLCCSA---GMSSLLVTKMEAALEGKIWAVSGDAVKTNIDQAD--VLLLGRYMLSSMKTLADERNVGIDVINPMHYDHALTLKK
3czcA	0.20	0.22	0.92	0.83	VKVLTAC-GNGMGSSMVIKMKVENALRIESASCSVGEAKGLASNYD--IVVASNHL---IHELDGRTNGKLIGLNLMD-DNEIKTKL
1f4pA	0.09	0.20	0.98	0.76	PKALIVYGSTT-GNTEYTAETIARELGYEVDSRDAASVEGLFEGFDLVLLGCSIPLFDSLEET-GAQGRKVACFGCGDFCGAVDAIE
3ecsA	0.19	0.26	0.92	0.69	FSVYVTESQPDLS-GKK-AKALCH-LNVPVTVVLDAAVGYI-EKADLVIVGAEGVVTNQ-AVCAKAQNKPFYVVAQQDVPDKFKY--
2l2qA	0.18	0.24	0.91	0.61	MNILLVC-----GAG-MLVQRIEKYANINIEAIAETRLSEVVDRFD--VVLLARFNKKRLEEITKPKGIPIEIINTIDYQLAINAFN
4dddA	0.14	0.20	0.97	0.74	KRVNIGSPG---TGVRVAMLKLLGEKGWMAELKSSEQAQALCDNKIDVMVDVIGHPNASIQEASATCDIKFIPLDDRLIDDLHAKYP
Amino acid sequence of Sll1961					ARVLVTVPQRDIGAGQLILNELEQALVIPVQLVPMETLKQTLSELPSGTVVTSRYFLAEAESIATPYDVRVIPVDIYDYSKELELVK

(d) Residues 223-327 of Sll1961

PDB	*Iden1*	*Iden2*	*Cov*	*Z*	Alignment
5vyrA	0.16	0.19	0.92	0.78	AIAPNTRVLVAGYGPAEFCVTTLIGMGVEIDKIAVATHEDNRNCGLHSMLRLRNIQFTTAAASEEFYEFGANFA------PDMIISMHYIPGRFLKLAKK--GSV
3gnsA	0.22	0.23	0.89	0.72	---ENKTYVIMSIAFGVAKVLD-----QLGAKLVFTYRKERSRKELEKLLEQAHLYQIDVQSDEEVINGFEQIGKDVGNIDGVYHSIQDISSYSLTIVAHE----
5hsgA	0.08	0.13	0.94	0.64	----GPTYALVQINALFFNLNKGAQDAASGKDLVIFNSN-DNPVAQNDAIENYKGILVAAIDVNGI-PAVKEAAAAIPGPQAAQVGDPEKGAEALNALNSITSGK
1kjqA	0.18	0.18	0.88	0.76	LRPAATRVMLL--GSGELGKEVAIECQRLGVEVIAVDRYADAPAMHVA----HRSHVINMLDGDALRRVVELEK------PHYIVPEIAIATDMLIQLEEE-GLN
4tklA	0.17	0.15	0.98	0.33	SDLKGKRILITGSTEGIGMATAIELAR-YGAVVGLNSVDPADPALLLGKLRDGAFFRADITKTAECQRLVSAFVERFDGIDVLINNAGGLANIDDAFYDRVMDL-
2pd6D	0.17	0.14	0.79	0.59	RL-RSA-LALVT-GSGIGRAVSVRLAG-EGATVAACDLDRAAAQETVRLLN-HAAFQADVSEARAARCLLEQVQACFSRPPSVVVSCA-----------------
4zhjA	0.16	0.21	0.94	0.67	ALNGRQLLKVVYVVSALSAAVRNINRTNSSLAIQLTGYLIENYANFKHDVSEANLFIASLIIEDLADKVVEAVTPYRDNLDAAIVFP------SMPQVMRLNKMG
5koiA	0.09	0.21	0.99	0.29	GLMQGKRGLIMGVNHSLAWGIAKQLA-AQGAELAFTYQGDALGKRVKPLAGSDFVLPCDVEDIATVDAVFEEIEKKWGGLDFLVHAIGFSDKTTRENFSRTMVIS
1e5dA	0.21	0.25	0.95	0.74	--PTN-KVVIFSMWHSTEKMARVLAESFRGCTVKLMWCKACHHSQIMSEISDAGAVIVGSPTHPYVAGTLQYIKG--LRPQNAFGSFGWSGESTKVLAEWLTGMG
Amino acid sequence of Sll1961					ALPENSCLGIVSLSPGILTIAEILIHSLRGESLFLKSALVSDPQKLRSLVRTARTIITDPASEPIVRQAIEAERHDLIRMPEIICSEHYIGEKSIAILKRELGLG

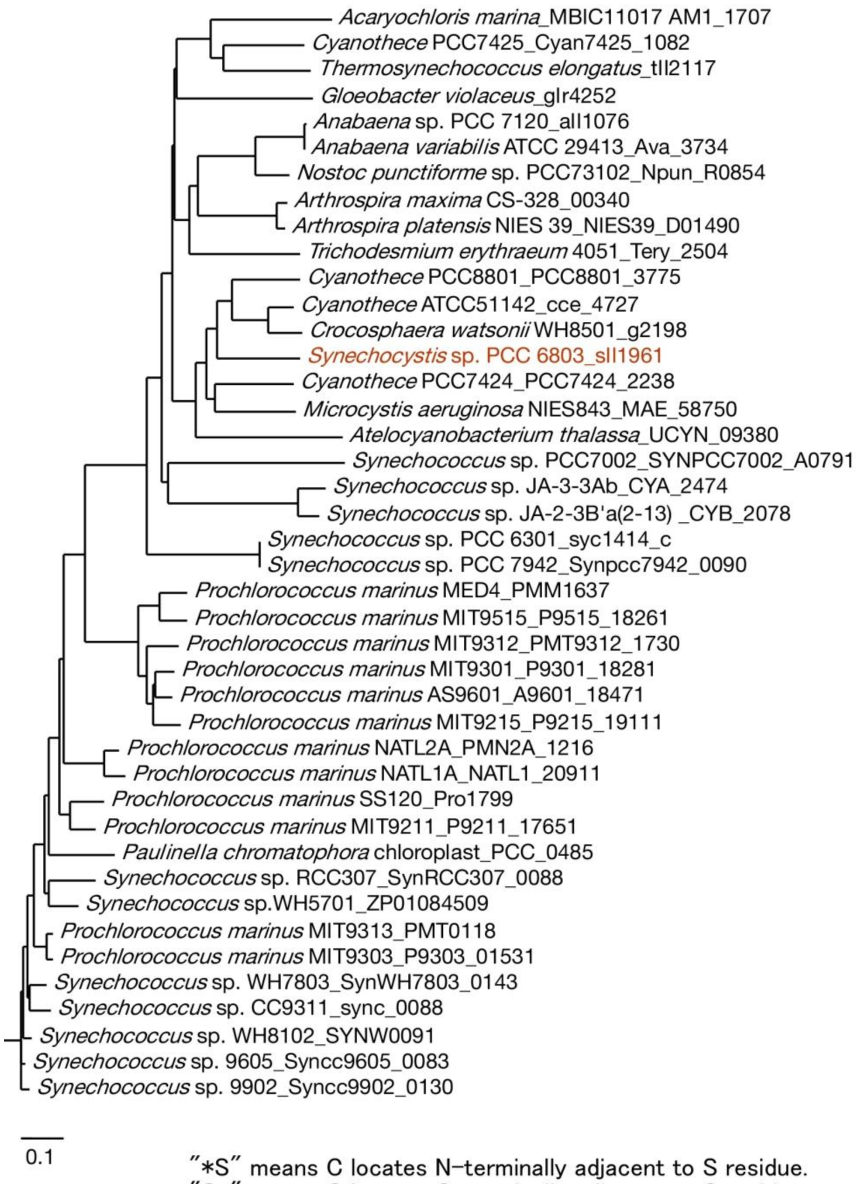

	23	107	124	134	229	280	307
*Acaryochloris marina*_MBIC11017 AM1_1707	C	S	F	A	R	C	C
Cyanothece PCC7425_Cyan7425_1082	S	S	L	A	C	S	C
*Thermosynechococcus elongatus*_tll2117	S	S	F	A	C	S	C
*Gloeobacter violaceus*_glr4252	S	S	F	C	C	T	S*
Anabaena sp. PCC 7120_all1076	R	A	F	C	C	C	K
Anabaena variabilis ATCC 29413_Ava_3734	R	A	F	C	C	C	K
Nostoc punctiforme sp. PCC73102_Npun_R0854	R	A	F	C	C	S	K
Arthrospira maxima CS-328_00340	R	S	F	C	Y	C	C
Arthrospira platensis NIES 39_NIES39_D01490	R	S	F	C	Y	C	C
Trichodesmium erythraeum 4051_Tery_2504	R	S	L	C	Y	C	C
Cyanothece PCC8801_PCC8801_3775	R	S	F	*S	R	S	C
Cyanothece ATCC51142_cce_4727	R	S	F	C	R	S	C
Crocosphaera watsonii WH8501_g2198	R	T	S	C	R	S	C
Synechocystis sp. PCC 6803_sll1961	R	T	C	S	C	T	C
Cyanothece PCC7424_PCC7424_2238	R	S	F	C	R	T	C
Microcystis aeruginosa NIES843_MAE_58750	R	S	L	C	R	S	C
*Atelocyanobacterium thalassa*_UCYN_09380	R	N	F	C	R	S	C
Synechococcus sp. PCC7002_SYNPCC7002_A0791	Q	A	F	C	C	C	P*
Synechococcus sp. JA-3-3Ab_CYA_2474	C	G	L	C	C	C	C
Synechococcus sp. JA-2-3B'a(2-13) _CYB_2078	C	G	L	C	C	C	C
Synechococcus sp. PCC 6301_syc1414_c	H	H	F	C	C	C	C
Synechococcus sp. PCC 7942_Synpcc7942_0090	H	H	F	C	C	C	C
Prochlorococcus marinus MED4_PMM1637	C	A	L	C	C	C	C
Prochlorococcus marinus MIT9515_P9515_18261	C	A	L	C	C	C	C
Prochlorococcus marinus MIT9312_PMT9312_1730	C	T	F	C	C	C	C
Prochlorococcus marinus MIT9301_P9301_18281	C	V	L	C	C	C	C
Prochlorococcus marinus AS9601_A9601_18471	C	V	L	C	C	C	C
Prochlorococcus marinus MIT9215_P9215_19111	C	A	L	C	S	C	C
Prochlorococcus marinus NATL2A_PMN2A_1216	C	S	F	C	C	C	C
Prochlorococcus marinus NATL1A_NATL1_20911	C	S	F	C	C	C	C
Prochlorococcus marinus SS120_Pro1799	C	S	F	C	C	C	C
Prochlorococcus marinus MIT9211_P9211_17651	C	S	F	C	C	C	C
Paulinella chromatophora chloroplast_PCC_0485	C	C	L	C	C	C	C
Synechococcus sp. RCC307_SynRCC307_0088	C	S	L	C	C	C	C
Synechococcus sp.WH5701_ZP01084509	C	C	L	C	C	C	C
Prochlorococcus marinus MIT9313_PMT0118	C	C	L	C	C	C	C
Prochlorococcus marinus MIT9303_P9303_01531	C	C	L	C	C	C	C
Synechococcus sp. WH7803_SynWH7803_0143	C	C	L	C	V	C	C
Synechococcus sp. CC9311_sync_0088	C	S	L	C	C	C	C
Synechococcus sp. WH8102_SYNW0091	C	C	L	C	C	C	C
Synechococcus sp. 9605_Syncc9605_0083	C	C	L	C	C	C	C
Synechococcus sp. 9902_Syncc9902_0130	C	C	L	C	C	C	C

"*S" means C locates N-terminally adjacent to S residue.
"S*" means C locates C-terminally adjacent to S residue.

Figure S1 Conservation of cysteine residues in Sll1961 orthologs.

Cysteine residues conserved in Sll1961 orthologs were shown with phylogenetic tree of Sll1961 orthologs. A set of amino acid sequences of Sll1961 orthologs was retrieved from Gclust Server (http://gclust.c.u-tokyo.ac.jp/) and the phylogenetic tree was generated using Phylodendron software (http://iubio.bio.indiana.edu/treeapp/treeprint-form.html). The numerals shown in the head line are the amino acid positions in Sll1961. "*S" and "S*" mean a cysteine residue locates N-terminally and C-terminally adjacent to the serine residue, respectively.

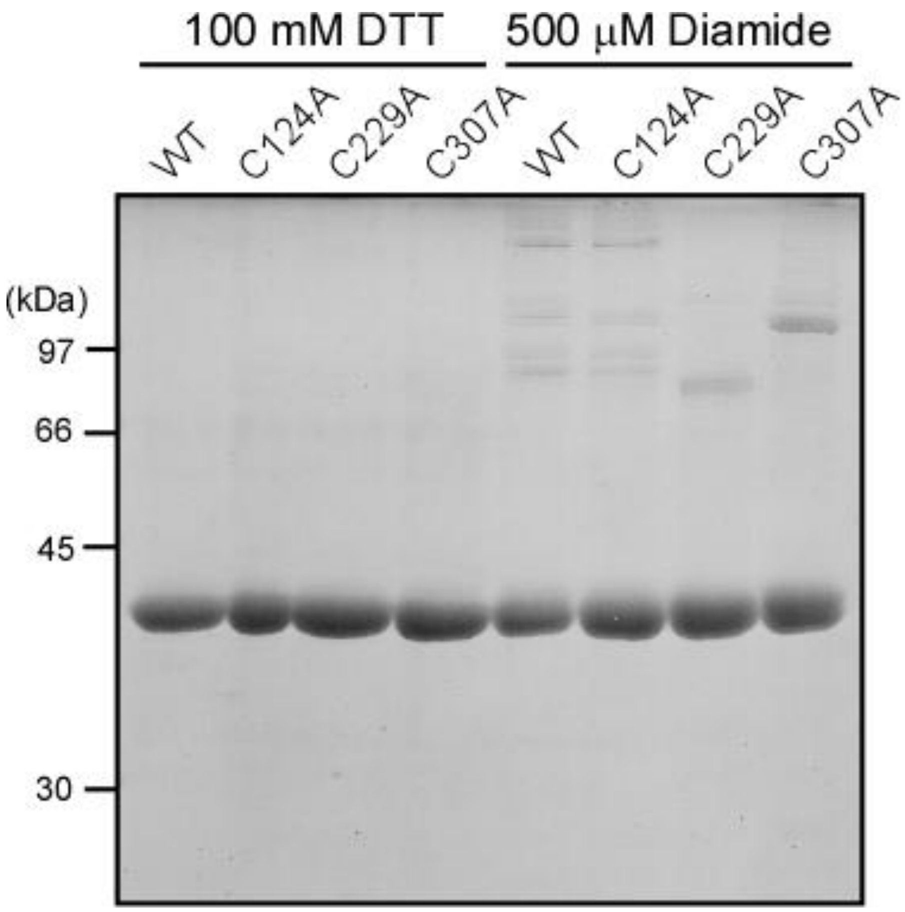

Figure S2 Redox treatment of WT and cysteine mutants of His-Sll1961 recombinant proteins.

His-Sll1961$_{WT}$, His-Sll1961$_{C124A}$, His-Sll1961$_{C229A}$ and His-Sll1961$_{C307A}$ proteins were treated with 100 mM DTT for 15 min or 500 μM diamide for 1 h and fractionated by non-reducing 12% SDS-PAGE.

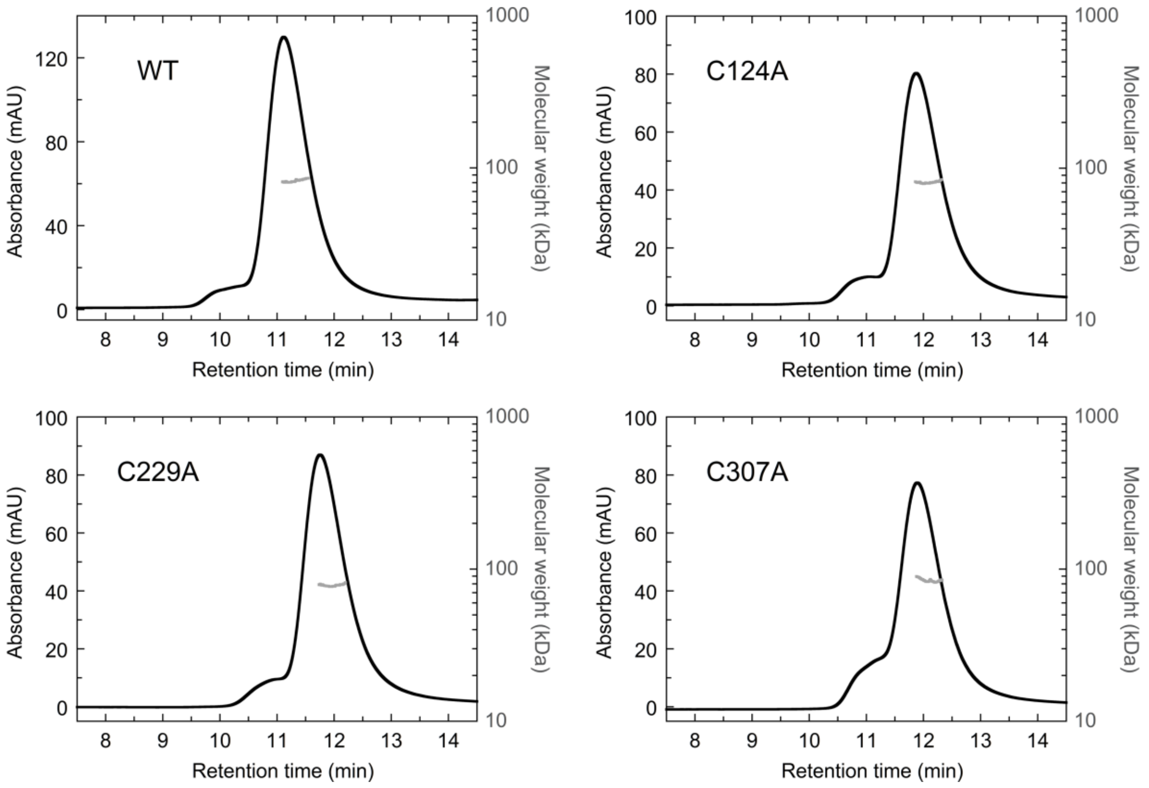

Figure S3 Elution profiles of size exclusion chromatography equipped with static light scattering.

WT and cysteine mutants of His-Sll1961 recombinant proteins were reduced with 100 mM DTT for 15 min at room temperature and applied to a gel filtration column. Molecular weights estimated by static light scattering are shown by gray lines (right axis).

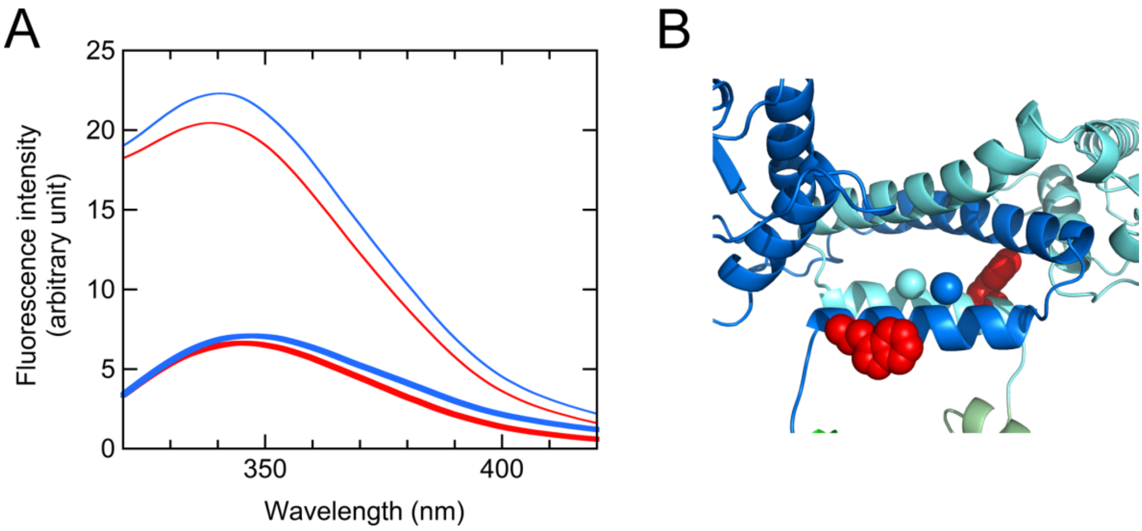

Figure S4 Fluorescence spectra of Sll1961.

(A) Red and blue curves show the fluorescence spectra of Sll1961 in the oxidized and reduced forms, respectively. Thick continuous lines show the spectra obtained by excitation at 295 nm, which indicate the fluorescence of a single tryptophan in Sll1961 (W130). Thin continuous lines show the spectra obtained by excitation at 280 nm, which indicate the fluorescence of both tryptophan and tyrosine residues. The peak wavelengths of the spectra obtained by excitation at 280 nm were 339 and 342 nm for the oxidized and reduced forms, while those obtained by excitation at 295 nm were 344 and 347 nm, respectively. It is known that when tryptophan residues are exposed to solvent, the peak wavelength of tryptophan fluorescence is ~350 nm, while it is ~330 nm when tryptophan residues are buried inside a protein molecule[35]. Thus, the present results indicate that W130 of Sll1961 is exposed to solvent, which is consistent with the model structure (see below). (B) Enlarged view of the structure of the N-terminal DBD in the Sll1961 homodimer predicted in this study. W130 (red spheres) is located at the N-terminal DBD and is 45% exposed to solvent. Blue and cyan spheres show the sulphur atoms of C124. The figure was drawn using the PyMOL Molecular Graphics System, Schrödinger, LLC.

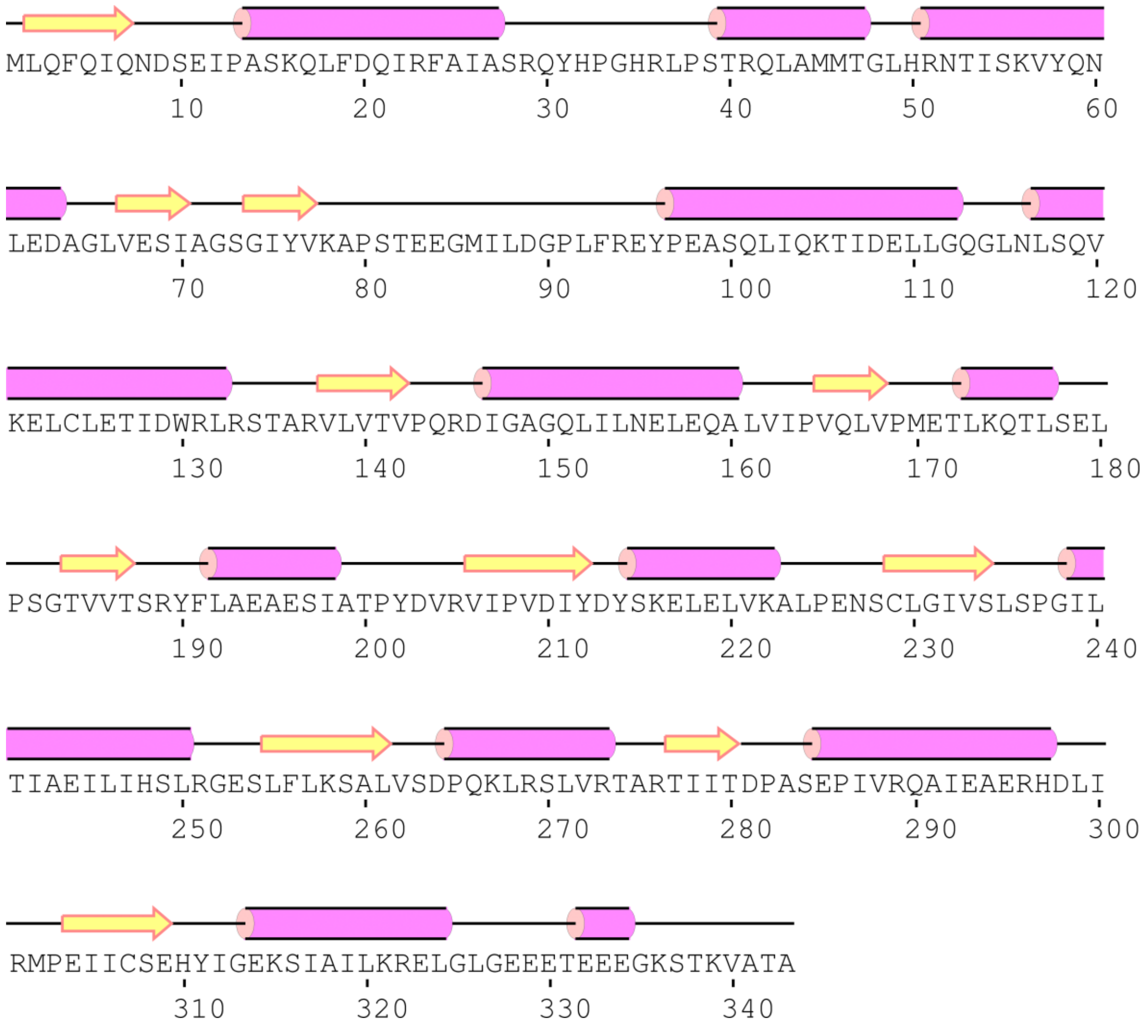

Figure S5 Secondary structure prediction of Sll1961.

Secondary structures predicted by PSIPRED[21] are shown along with the amino-acid sequence of Sll1961. Pink cylinders and yellow arrows denote α-helix and β-strand, respectively.

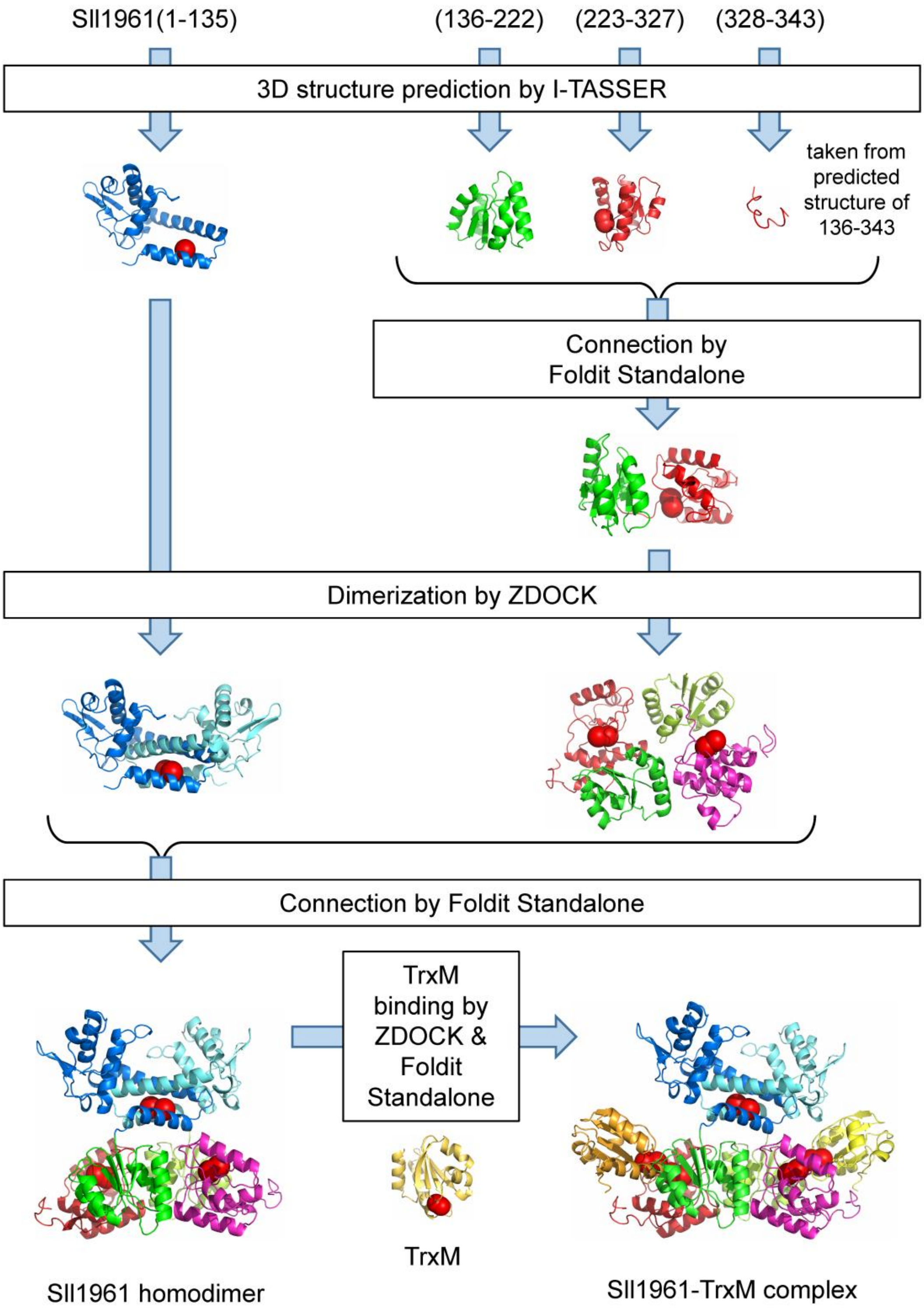

Figure S6 Procedure for modeling tertiary structures of Sll1961 and the Sll1961-TrxM complex.

Red spheres show cysteine residues. The figure was drawn using the PyMOL Molecular Graphics System, Schrödinger, LLC.
